# Is posture-related craniospinal compliance shift caused by jugular vein collapse? A theoretical analysis

**DOI:** 10.1186/s12987-017-0053-6

**Published:** 2017-02-16

**Authors:** Manuel Gehlen, Vartan Kurtcuoglu, Marianne Schmid Daners

**Affiliations:** 1 Institute for Dynamic Systems and Control, Department of Mechanical and Process Engineering, ETH Zurich, Zurich, Switzerland; 2 The Interface Group, Institute of Physiology, University of Zurich, Zurich, Switzerland; 3 Neuroscience Center Zurich, and the Zurich Center for Integrative Human Physiology, University of Zurich, Zurich, Switzerland; 4 Product Development Group Zurich, Department of Mechanical and Process Engineering, ETH Zurich, Zurich, Switzerland

**Keywords:** Craniospinal, Compliance, Cerebrospinal fluid dynamics, Posture

## Abstract

**Background:**

Postural changes are related to changes in cerebrospinal fluid (CSF) dynamics. While sitting up leads to a decrease in cranial CSF pressure, it also causes shifts in the craniospinal CSF volume and compliance distribution. We hypothesized that jugular vein collapse in upright posture is a major contributor to these shifts in CSF volume and compliance.

**Methods:**

To test this hypothesis, we implemented a mathematical lumped-parameter model of the CSF system and the relevant parts of the cardiovascular system. In this model, the CSF and the venous system are each divided into a cranial and a spinal part. The pressures in these cranial and spinal portions differ by the posture-dependent hydrostatic pressure columns in the connecting vessels. Jugular collapse is represented by a reduction of the hydrostatic pressure difference between cranial and spinal veins. The CSF pressure–volume relationship is implemented as a function of the local CSF to venous pressure gradient. This implies that an increase in CSF volume leads to a simultaneous displacement of blood from adjacent veins. CSF pulsations driven by the cardiovascular system are introduced through a pulsating cranial arterial volume.

**Results:**

In upright posture, the implemented CSF pressure–volume relationship shifts to lower cranial CSF pressures compared to the horizontal position, leading to a decrease in cranial CSF pressure when sitting up. Concurrently, the compliance of the spinal compartment decreases while the one of the cranial compartment increases. With this, in upright posture only 10% of the CSF system’s compliance is provided by the spinal compartment compared to 35% in horizontal posture. This reduction in spinal compliance is accompanied by a caudal shift of CSF volume. Also, the ability of the spinal CSF compartment to compensate for cerebral arterial volume pulsations reduces in upright posture, which in turn reduces the calculated craniospinal CSF flow pulsations.

**Conclusion:**

The mathematical model enabled us to isolate the effect of jugular collapse and quantify the induced shifts of compliance and CSF volume. The good concordance of the modelled changes with clinically observed values indicates that jugular collapse can be considered a major contributor to CSF dynamics in upright posture.

## Background

Several pathologies of the central nervous system, like hydrocephalus and syringomyelia, are caused or characterized by altered cerebrospinal fluid (CSF) dynamics. Therefore, the treatment of these conditions typically aims at restoring physiological circulation of CSF and requires profound knowledge of the underlying pathophysiology. However, CSF dynamics are mostly studied in horizontal posture, even though we spend most of our time upright and CSF dynamics fundamentally change with posture. For example, sitting up not only leads to changes in intracranial pressure (ICP), but also to a caudal shift of CSF volume and an inversion of the compliance distribution between the cranial and the spinal part of the CSF system. This inversion of the craniospinal compliance was first observed by Magnaes in a small number of subjects [1]. In a recent study by Alperin et al. [2], the pulse amplitude of craniospinal CSF flow recorded with magnetic resonance imaging (MRI), decreased in sitting posture, which supports the findings of Magnaes. In CSF shunts, anti-siphon devices are used to counteract posture-related changes in pressures. However, the diversity of functional principles on which these devices are based, indicates that the mechanisms of the posture-related changes in CSF dynamics and their link to hemodynamics are largely unknown [2]. Knowing the causalities of these interactions would contribute to the understanding of individual pathologies and to the choice of the most appropriate treatment option, especially in the context of various comorbidities typically seen in these patients.

What we do know is that CSF pressure in equilibrium conditions is a function of venous pressure through Davson’s equation [3], and that at least cranial venous pressure changes with posture due to hydrostatic gradients along the blood vessels. Also, cranial venous pressure changes with the state of the jugular veins: when they collapse in upright posture venous resistance increases, reducing both the posture-related decrease in cranial venous pressure and in CSF pressure [4–6].

We hypothesized that the collapse of the jugular veins when upright not only affects mean ICP, but that it also causes the aforementioned caudal shift of CSF volume: interruption of the venous hydrostatic pressure column decreases the cranial CSF to venous pressure gradient by diminishing the reduction in cranial venous pressure when sitting up. Due to the exponential nature of the CSF system’s pressure–volume relationship [7], this in turn, increases cranial compliance in upright posture. At the same time, the non-interrupted hydrostatic pressure column leads to an increased CSF to venous pressure gradient below the level of the jugular veins, causing the observed caudal shift of CSF volume. Consequently, the spinal dural sac volume increases, reducing the compliance of the spinal CSF space [1].

We aimed at testing this hypothesis by implementing a mathematical model of the CSF system and the relevant parts of the cardiovascular system. This has enabled us to isolate the effect of jugular vein collapse and quantify the induced shifts of compliance and CSF volume. These estimated changes in CSF dynamics were then compared to the measurements of Magnaes [1]. Testing the hypothesis without a mathematical model would be difficult, as jugular collapse can hardly be avoided in vivo. To allow for further model validation, we computed changes in craniospinal CSF flow secondary to changes in craniospinal compliance distribution. Unlike the distribution of compliance itself, changes in CSF flow can be easily measured with MRI and used as surrogate for changes in compliance distribution. With this, we were able to validate the model by comparing the craniospinal flow rates estimated by the model to reported flow rates recorded in supine and sitting posture [2, 8, 9].

## Methods

**Fig. 1:**
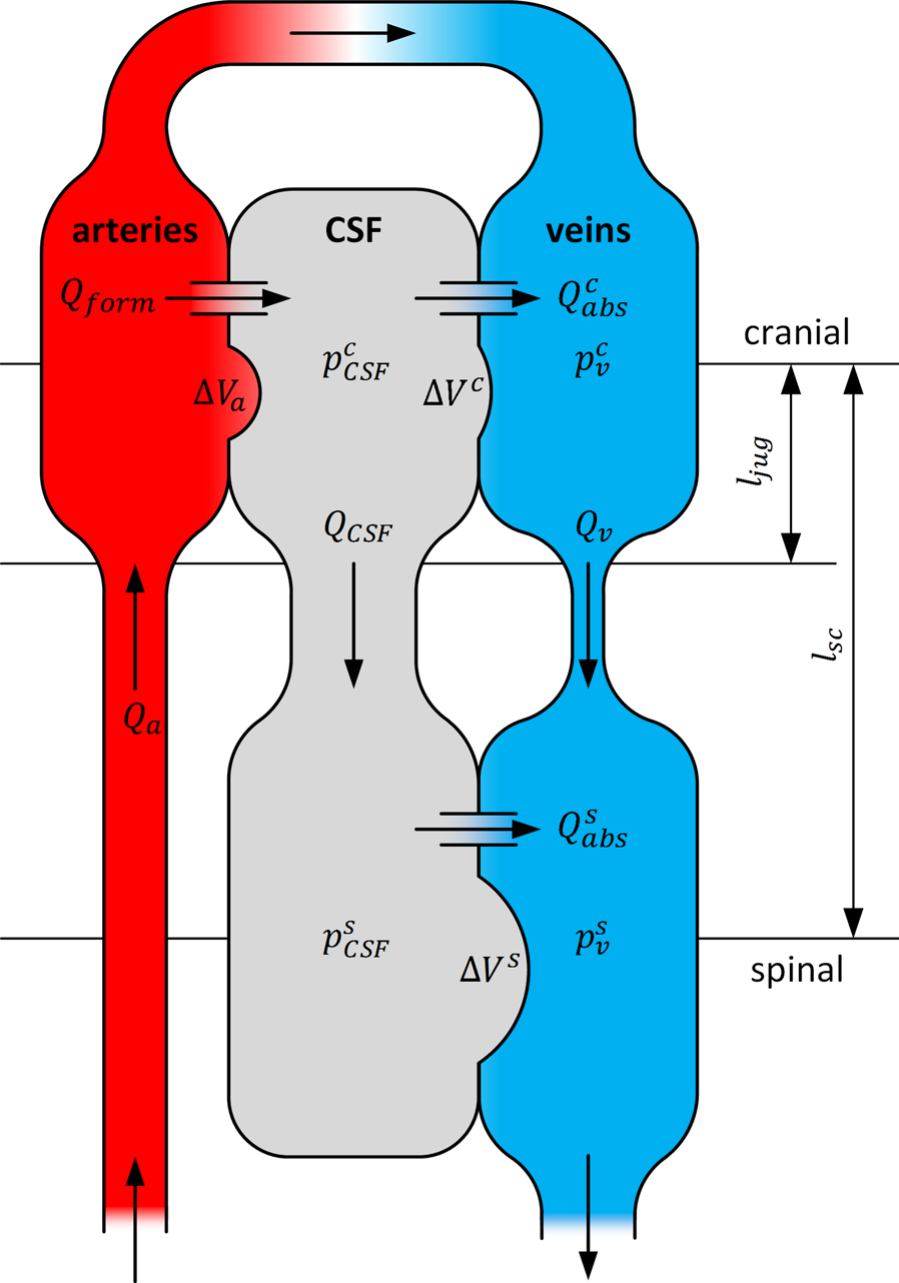
Model schematic: interaction between CSF and cardiovascular systems in upright posture. In the mathematical model, CSF and venous blood are divided into cranial and spinal compartments. The corresponding pressures at the cranial ($p_{CSF}^{c}$ and $p_{v}^{c}$) and spinal ($p_{CSF}^{c}$ and $p_{v}^{c}$) level differ by hydrostatic pressure columns that are characterized by the distances $l_{sc}$ and $l_{jug}$. The interaction of CSF and venous blood is determined by the local pressure–volume relationships ($\Delta V^{c}$ and $\Delta V^{s}$). The pulsating arterial blood flow $Q_{a}$ leads to a pulsating change in the cranial arterial blood volume $\Delta V_{a}$, which is compensated by craniospinal flows of CSF and venous blood ($Q_{CSF}$ and $Q_{v}$). CSF formation ($Q_{form}$) and absorption ($Q_{abs}$) are also indicated

We used a lumped parameter description of the interaction between the CSF and cardiovascular systems as shown in Fig. 1. As most of the CSF system’s compensatory reserve is provided by concurrent venous volume adaptation, the CSF pressure–volume relationship was implemented as a function of the local CSF to venous pressure gradient [10, 11]. This implies that an increase in CSF volume leads to a simultaneous displacement of venous blood from adjacent veins. In upright posture, hydrostatic pressure gradients between different locations in the CSF space and in the venous system become relevant. In our model, the CSF space and the venous compartment were divided into a cranial and a spinal portion, and CSF and venous pressures were evaluated at these two locations. While in horizontal posture these cranial and spinal pressures are approximately equal, they differ in upright posture due to the hydrostatic gradients in the connecting vessels. If the jugular veins did not collapse in upright posture, spinal and cranial venous pressures would differ by the same hydrostatic pressure column as spinal and cranial CSF pressure, respectively. However, in upright posture and for reasonably low central venous pressures, this hydrostatic pressure gradient is interrupted by the collapsing jugular veins [4, 5]. The collapsed segment of the jugular veins acts as a differential pressure valve with opening pressure equal to ambient pressure. Therefore, in upright posture, cerebral venous outflow is partly redirected through the high-resistance pathway presented by the vertebral veins, and venous pressure at the site of the collapse, is regulated to ambient pressure by the jugular veins [4]. Thus, cerebral venous pressure is only determined by the hydrostatic pressure gradient above the site of the collapse [4, 5]. The reference level of the spinal portion was chosen such that the spinal venous pressure is independent of posture. In contrast to the venous blood vessels, the hydrostatic column in the CSF system was assumed to be uninterrupted.

Cerebrospinal fluid pulsations driven by the cardiovascular system were accounted for through a pulsating cranial arterial volume. The pulsations of this arterial volume were based on recorded flow rates in the internal carotid and vertebral arteries. As CSF competes with the arterial pulsations for the available compliance, the arterial volume was added to the cranial CSF volume. Instantaneous flow rates for CSF and venous blood between cranial and spinal compartments were calculated based on a volume balance, assuming constant cranial volume (Monroe–Kelly doctrine).

### Model derivation

#### Local pressure–volume relationships

The pressure gradient $\Delta p$ between the CSF pressure $p_{CSF}$ and the venous pressure $p_{v}$ required for the displacement of venous blood was described by an exponential function:

1 $$\Delta p = p_{CSF} - p_{v} = p_{1} \cdot e^{E \cdot \Delta V} + p_{0} ,$$

where $\Delta V$ is the CSF volume increase from baseline (supine equilibrium), and $E$, $p_{1}$, and $p_{0}$ are constants (Table 1). This exponential function was derived from the exponential pressure–volume relation originally described by Marmarou [7, 12, 13]:

**Table 1:** Parameters, distinctive for normal pressure hydrocephalus

Parameter	Symbol	Value	Unit	Reference
Elastance	$E$	0.23	mL^−1^	[9, 16]*
Exponential parameter	$p_{1}$	4	mmHg	[16]*
Offset pressure	$p_{0}$	3.2	mmHg	[16]*
Rate of CSF formation	$Q_{form}$	0.35	mL/min	[13]
Total CSF outflow resistance	$R_{abs}^{tot}$	20.6	mmHg/(mL/min)	[13, 16]*
Relative spinal compliance	$k_{V}$	0.35	–	[17]
Spinal venous pressure	$p_{v}^{s}$	5.3	mmHg	[4]
Distance between spinal and cranial reference points	$l_{sc}$	33.8	cm	[4]
Distance between jugular veins and cranial reference point	$l_{jug}$	11.0	cm	[4]
Density of CSF	$\rho_{CSF}$	1000	kg/m^3^	
Density of blood	$\rho_{blood}$	1060	kg/m^3^	
Gravitational acceleration	$g$	9.81	m/s^2^	
Relative spinal outflow conduction	$k_{R}$	n/a^#^	–	

* Computed from data given in the referenced work: $E = \ln \left( {RPPC + 1} \right)/\Delta V_{a}$ [18], $p_{1} = ICP_{r} - p_{0} - p_{v}^{s}$, $p_{0} = p_{0}^{M} - p_{v}^{s}$, $R_{abs}^{tot} = (ICP_{r} - p_{v}^{s} )/Q_{form}$ [3]

^#^ The value of this parameter is unknown. It is estimated in this study

2 $$p_{CSF} = p_{1} \cdot e^{E \cdot \Delta V} + p_{0}^{M} ,$$

where the reference pressure $p_{0}^{M}$ is the sum of the postural pressure component $p_{0}$ and the venous pressure $p_{v}^{s}$ [11, 14]. Describing the pressure–volume relation as a function of the CSF to venous pressure gradient, $\Delta p$, allows changes in venous pressure to be accounted for [10, 11]. In the model, the CSF space and the venous compartment were divided into a cranial and a spinal part. Thus, an increase in CSF volume can be compensated by a displacement of venous blood from the cranium ($\Delta V^{c}$) or from venous vessels adjacent to the spinal canal and the spinal thecal sac ($\Delta V^{s}$):

3 $$\Delta V^{tot} = \Delta V^{c} + \Delta V^{s} .$$

In horizontal posture, where cranial and spinal CSF as well as venous pressures can be assumed equal ($p_{CSF}^{c} = p_{CSF}^{s}$ and $p_{v}^{c} = p_{v}^{s}$), the combined pressure–volume relationship $\Delta V^{tot}$ must be equal to the established relation (Eq. 2). Thus, the local pressure–volume relationships in the spinal and the cranial compartment were implemented as in Eq. 1, but as functions of the respective local CSF to venous pressure gradients:

4 $$\Delta V^{c} \left( {p_{CSF}^{c} } \right) = \left( {1 - k_{V} } \right) \cdot \frac{1}{E} \cdot \ln \left( {\frac{{p_{CSF}^{c} - p_{v}^{c} - p_{0} }}{{p_{1} }}} \right)$$

5 $$\Delta V^{s} \left( {p_{CSF}^{s} } \right) = k_{V} \cdot \frac{1}{E} \cdot \ln \left( {\frac{{p_{CSF}^{s} - p_{v}^{s} - p_{0} }}{{p_{1} }}} \right).$$

Here, $k_{V}$ is a constant that describes the portion of the total compensatory reserve of the CSF system attributed to the spinal compartment. In horizontal posture, $k_{v}$ is the spinal compliance contribution as measured by Magnaes [1].

#### Hydrostatic pressure gradients

In upright posture, spinal CSF pressure $p_{CSF}^{s}$ is higher than the cranial CSF pressure $p_{CSF}^{c}$ due to the hydrostatic pressure column of length $l_{sc}$:

6 $$p_{CSF}^{s} = p_{CSF}^{c} + \rho_{CSF} \cdot g \cdot l_{sc} ,$$

where $l_{sc}$ is the vertical distance between the reference points of the spinal and the cranial compartments (Fig. 1), $\rho_{CSF}$ is CSF density, and $g$ is gravitational acceleration. $p_{CSF}^{c}$ is often referred to as ICP. If lumbar CSF pressure is measured, Eq. 6 is accounted for by sensor calibration [4].

For the spinal part of the model, the hydrostatic indifference point of the venous system was chosen as the reference location. Thus, the spinal venous pressure $p_{v}^{s}$ was assumed to be independent of posture.

Without collapse of the jugular veins, cranial and spinal venous pressures would also differ by a hydrostatic column of length $l_{sc}$:

7 $$p_{v}^{c} = p_{v}^{s} - \rho_{blood} \cdot g \cdot l_{sc} .$$

However, since venous pressure is equal to ambient pressure at the location where the jugular veins collapse, cerebral venous pressure is determined by the hydrostatic pressure gradient of length $l_{jug}$ instead [4, 5]:

8 $$p_{v}^{c} = - \rho_{blood} \cdot g \cdot l_{jug}$$

$l_{jug}$ is the distance between the upper end of the jugular collapse and the reference point of the cranial compartment.

Using Eqs. 6 and 8, the CSF pressure–volume relationships (Eqs. 4, 5) can also be written, for the upright posture, as a function of only the cranial CSF pressure ($p_{CSF}^{c}$):

9 $$\left. {\Delta V^{c} \left( {p_{CSF}^{c} } \right)} \right|_{up} = \left( {1 - k_{V} } \right) \cdot \frac{1}{E} \cdot \ln \left( {\frac{{p_{CSF}^{c} + \rho_{blood} \cdot g \cdot l_{jug} - p_{0} }}{{p_{1} }}} \right)$$

10 $$\left. {\Delta V^{s} \left( {p_{CSF}^{c} } \right)} \right|_{up} = k_{V} \cdot \frac{1}{E} \cdot \ln \left( {\frac{{p_{CSF}^{c} + \rho_{CSF} \cdot g \cdot l_{sc} - p_{v}^{s} - p_{0} }}{{p_{1} }}} \right).$$

#### Compliance

Compliance $C$ is defined as change of volume relative to the corresponding change in pressure [7]. It was calculated analytically as the slope of the pressure–volume curves (Eqs. 4, 5, 9, 10):

11 $$\left. {C^{c} \left( {p_{CSF}^{c} } \right)} \right|_{hor} = \left. {\frac{{d\Delta V^{c} }}{{dp_{CSF}^{c} }}} \right|_{hor} = \frac{{1 - k_{V} }}{E} \cdot \frac{1}{{p_{CSF}^{c} - p_{v}^{s} - p_{0} }}$$

12 $$\left. {C^{s} \left( {p_{CSF}^{c} } \right)} \right|_{hor} = \left. {\frac{{d\Delta V^{s} }}{{dp_{CSF}^{c} }}} \right|_{hor} = \frac{{k_{V} }}{E} \cdot \frac{1}{{p_{CSF}^{c} - p_{v}^{s} - p_{0} }}$$

13 $$\left. {C^{c} \left( {p_{CSF}^{c} } \right)} \right|_{up} = \left. {\frac{{d\Delta V^{c} }}{{dp_{CSF}^{c} }}} \right|_{up} = \frac{{1 - k_{V} }}{E} \cdot \frac{1}{{p_{CSF}^{c} + \rho_{blood} \cdot g \cdot l_{jug} - p_{0} }}$$

14 $$\left. {\left. {C^{s} \left( {p_{CSF}^{c} } \right)} \right|_{up} = \frac{{d\Delta V^{s} }}{{dp_{CSF}^{c} }}} \right|_{up} = \frac{{k_{V} }}{E} \cdot \frac{1}{{p_{CSF}^{c} + \rho_{CSF} \cdot g \cdot l_{sc} - p_{v}^{s} - p_{0} }}.$$

#### CSF formation and absorption

The rate of CSF formation $Q_{form}$ was implemented as posture independent and constant [13] (Table 1). The cranial and the spinal CSF absorption rates were assumed to be proportional to the local CSF to venous pressure gradient, $\Delta p^{i}$:

15 $$Q_{abs}^{i} (\Delta p^{i} ) = \left( {p_{CSF}^{i} - p_{v}^{i} } \right)/R_{abs}^{i}$$

where $R_{abs}^{i}$ is the local CSF outflow resistance. While the overall outflow resistance $R_{abs}^{tot}$ can be determined clinically [15], its craniospinal distribution characterized by the coefficient, $k_{R}$, is generally unknown.

16 $$\frac{1}{{R_{abs}^{tot} }} = \underbrace {{k_{R} /R_{abs}^{tot} }}_{{1/R_{abs}^{s} }} + \underbrace {{\left( {1 - k_{R} } \right)/R_{abs}^{tot} }}_{{1/R_{abs}^{c} }}$$

#### Model parameters

The parameters used for the calculations in this study (Table 1) are characteristic for patients with normal pressure hydrocephalus (NPH). They describe a patient with 12.5 mmHg resting intracranial pressure ($ICP_{r}$).

#### Sensitivity analysis

To analyze the sensitivity of the investigations with respect to the employed parameter values, a three step sensitivity analysis was performed. First, all calculations were repeated with a second parameter set (*E* = 0.1/mL, *p*_1_ = 10 mmHg, *p*_0_ = −5.3 mmHg, $R_{abs}^{tot} = 13.4\,{\text{mmHg}}/({\text{mL}}/\hbox{min} )$) that describes physiological CSF dynamics [19]. Second, the parameters determining the hydrostatic gradients within the CSF and the venous system ($l_{sc}$, $l_{jug}$, and $p_{v}^{s}$) were varied within reported standard deviations (*l*_sc_ = 33.8 ± 2.5 cm, $p_{v}^{s} = 5.3 \pm 2.5\, {\text{mmHg}}$) [4] one at a time. Third, the compliance distribution assumed in horizontal position was varied by ±50% ($k_{V} = 0.35 \pm 0.175$).

#### Cranial arterial volume

Given that arterial pressure is substantially higher than CSF pressure in all but the most extreme pathologic conditions, arterial blood flow rate to the cranium $Q_{a}$ was assumed unaffected by CSF dynamics. Therefore, the change in cranial arterial volume can be derived from in vivo measurements of $Q_{a}$. We used flow rates recorded by phase-contrast MRI in the internal carotid and vertebral arteries as arterial blood flow, $Q_{a}$. These flow rates were obtained from the average of 16 NPH patients [9]. Additionally, the flow rates of a healthy volunteer in supine and sitting position [2] were applied to validate the predicted changes in craniospinal CSF flow.

The flows leaving the cranial arterial compartment are the capillary blood flow and the rate of CSF formation, both of which were assumed non-pulsatile. Therefore, the volume balance in the cranial arterial compartment reduces to

17 $$\Delta V_{a} \left( t \right) = \mathop \int \limits_{0}^{t} Q_{a} \left( t \right) - \bar{Q}_{a} dt ,$$

where $\bar{Q}_{a}$ is the mean arterial flow rate over one cardiac cycle.

### Evaluation

#### Sitting up

Immediately upon sitting up, total CSF volume is identical to the equilibrium volume in horizontal position. In other words, the change in total CSF volume is initially zero. The corresponding CSF pressure in upright posture was determined by numerically solving

18 $$\left. {\Delta V^{tot} \left( {p_{CSF}^{c} } \right)} \right|_{up} \mathop = \limits^{!} \left. {\Delta V^{tot} \left( {p_{CSF}^{c} } \right)} \right|_{hor} = 0$$

for the cranial CSF pressure $p_{CSF}^{c}$. While total CSF volume will not change immediately after changing posture, a rapid caudal shift of CSF volume through the unrestricted CSF pathways can be expected. This shifted volume corresponds to the change in spinal CSF volume $\Delta V^{s}$ (Eq. 10) evaluated at the above calculated CSF pressure.

#### Upright equilibrium

The upright equilibrium is reached when CSF absorption and formation rates are equal. However, the pressure-dependent CSF absorption rate can only be calculated for a known craniospinal absorption distribution ($k_{R}$). Thus, Magnaes’ observation [1] of unchanged total compliance in upright posture was used to determine CSF pressure in upright equilibrium:

19 $$\left. {C^{tot} \left( {p_{CSF}^{c} } \right)} \right|_{up} \mathop = \limits^{!} \left. {C^{tot} \left( {p_{CSF}^{c} } \right)} \right|_{hor}$$

Then, the ratio $k_{R}$ that leads to equal CSF formation and absorption at this CSF pressures was calculated:

20 $$Q_{form} \mathop = \limits^{!} \frac{{p_{CSF}^{c} - p_{v}^{c} }}{{R_{abs}^{c} }} + \frac{{p_{CSF}^{s} - p_{v}^{s} }}{{R_{abs}^{s} }} = \frac{{k_{R} }}{{R_{abs}^{tot} }}\left( {p_{CSF}^{c} - p_{v}^{c} } \right) + \frac{{1 - k_{R} }}{{R_{abs}^{tot} }}\left( {p_{CSF}^{s} - p_{v}^{s} } \right).$$

#### Simulation of craniospinal flow rates

Any increase or decrease in volume of one entity (fluids and tissue) within the cranium has to be compensated, respectively, by an equivalent volume decrease or increase of the other entities (Monroe–Kelly doctrine) [20]. Therefore, the pulsating volume increase of arterial blood in the cranium $\Delta V_{a}$ (Eq. 17) has to be compensated by a reduction in CSF or venous blood volume. Similar to changes in cranial CSF volume, a change in the cranial arterial volume can be compensated by either a shift of CSF from or to the spinal compartment or a reduction or increase of cranial venous blood volume. With this, cranial arterial blood directly competes with CSF for the available compliance, and the total amount of displaced venous blood $\Delta V^{tot}$ is equal to the sum of changes in CSF volume $\Delta V_{CSF}$ and arterial volume $\Delta V_{a} \left( t \right)$:

21 $$\Delta V_{CSF} \left( t \right) + \Delta V_{a} \left( t \right)\mathop = \limits^{!} \Delta V^{tot} \left( {p_{CSF}^{c} } \right)$$

Solving this equation for the cranial CSF pressure $p_{CSF}^{c}$ allows for determining the current pressure-dependent CSF absorption rates and calculating the CSF volume $\Delta V_{CSF}$, which may fluctuate throughout a cardiac cycle:

22 $$\frac{d}{dt}\Delta V_{CSF} \left( t \right) = Q_{form} - Q_{abs}^{c} \left( {p_{CSF}^{c} } \right) - Q_{abs}^{s} \left( {p_{CSF}^{c} } \right).$$

The CSF volume and the cranial CSF pressure during a cardiac cycle were computed by solving this system of differential–algebraic equations (Eqs. 21, 22) using the Matlab (The MathWorks, Inc., Natick, MA, USA) variable-order solver ‘ode15s’.

Based on these computations, the CSF flow rate into the spinal compartment was calculated as the change in spinal CSF volume:

23 $$Q_{CSF} \left( t \right) = \frac{d}{dt}\Delta V^{s} \left( {p_{CSF}^{c} \left( t \right)} \right),$$

and the craniospinal venous flow rate was calculated based on a volume balance in the cranium:

24 $$Q_{v} \left( t \right) = Q_{a} \left( t \right) - Q_{CSF} \left( t \right).$$

## Results

After calculating the local and total pressure–volume relationships of the CSF space in horizontal and upright posture, these correlations were used to derive the local and total compliances. Based on this, CSF volume and pressure in upright posture were determined under the assumption of unchanged CSF volume (Eq. 18) or under the assumption of unchanged total compliance (Eq. 19). The posture-related volume and compliance shifts were then evaluated under these two conditions. Finally, the model output was calculated (Eqs. 21, 22) for one cardiac cycle and the craniospinal flow rates of blood and CSF were derived (Eqs. 23, 24) as a basis for discussion of model validity.

### Pressure–volume relationships

**Fig. 2:**
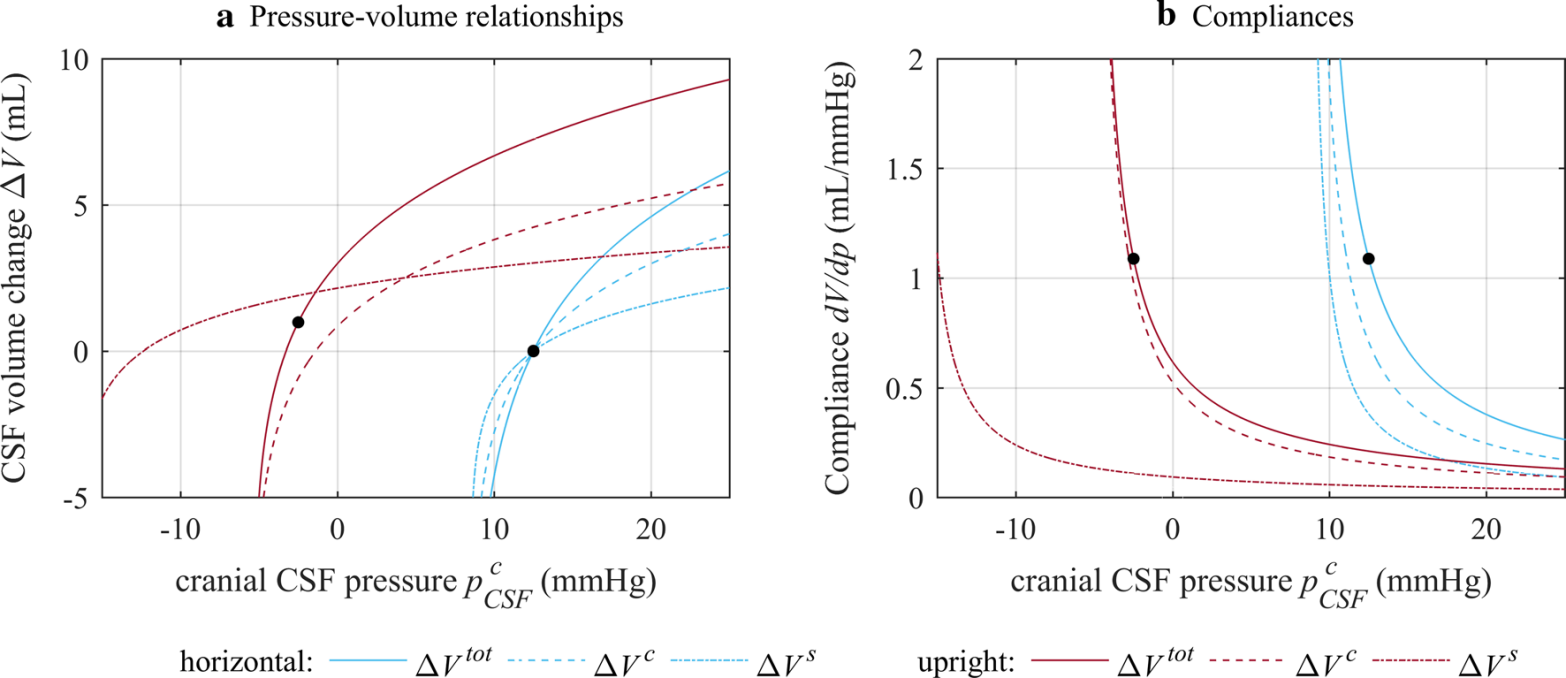
CSF pressure–volume relationships and compliances in horizontal and upright posture. **a** The cranial and spinal pressure–volume relationships $\Delta V^{c} (p_{CSF}^{c} )$ and $\Delta V^{s} (p_{CSF}^{c} )$ are plotted along with the combined overall pressure–volume relationship, $\Delta V^{tot} \left( {p_{CSF}^{c} } \right)$. **b** Visualizes the derivatives of these correlations (Eqs. 11–14), which represent the respective local and total compliances of the CSF system. Equilibrium conditions in both postures are indicated by *black dots*

In horizontal position, cranial and spinal CSF and venous pressures are equal. The overall pressure–volume relationship was thus described by Eq. 1. In upright posture, the local pressure–volume relationships shifted to lower cranial CSF pressures (Eqs. 9, 10) compared to the horizontal position (Fig. 2a). Hereby, the shift of the spinal pressure–volume relationship $\Delta V^{s} \left( {p_{CSF}^{c} } \right)$was determined by the distance between the spinal and the cranial reference point, $l_{sc}$. With the assumption that the jugular veins collapse in upright posture, the shift of the cranial pressure–volume relationship ($\Delta V^{c} \left( {p_{CSF}^{c} } \right)$) is reduced to a hydrostatic pressure column of length $l_{jug}$. The overall pressure–volume relationship $\Delta V^{tot} \left( {p_{CSF}^{c} } \right)$ was found by summation of these two local pressure–volume relationships (Eq. 3).

### Compliance

In Fig. 2b, the local compliances derived analytically from the corresponding pressure–volume relationships (Eqs. 11–14) were plotted along with the combined total compliance for horizontal and upright posture. Similar to the total pressure–volume relationship, the total compliance shifted towards lower cranial CSF pressures in upright posture. Due to a steep increase of the cranial compliance at low CSF pressures, the cranial compartment became the dominant source of compliance at cranial CSF pressures below approximately 0 mmHg.

### Posture change

The resulting cranial CSF pressure in upright posture without any change in CSF volume, which corresponds to the conditions directly after sitting up from horizontal position, was −3.3 mmHg. This decrease in cranial CSF pressure was accompanied by a shift of CSF from the cranial to the spinal compartment ($\Delta V^{s}$ in Table 2). The condition of equal compliance in horizontal and upright posture was satisfied for a cranial CSF pressure of −2.5 mmHg as depicted in Fig. 2b. It required a slight increase in total CSF volume (Table 2). Despite this increase in total CSF volume, the amount of cranial CSF was smaller than in horizontal equilibrium. Furthermore, in equilibrium, the rate of CSF absorption has to match the rate of formation. In upright posture, this was achieved for *k*_R_ = 0.10. In other words, the spinal resistance to CSF outflow was around nine times the cranial resistance to CSF outflow (Eq. 16). While this meant that in horizontal position 10% of the CSF absorption occurred within the spinal compartment, it equated to 24% spinal absorption in upright posture due to the increased CSF to venous pressure gradient in the spinal compartment.

**Table 2:** Comparison of CSF pressure, volume and compliance in horizontal and upright posture

Posture condition	Horizontal	Upright	
	–	Eq. 18	Eq. 19
$p_{CSF}^{c}$ (mmHg)	12.5	−3.3	−2.5
$\Delta V^{tot}$ (mL)	0.0	0	1.0
$\Delta V^{c}$ (mL)	0.0	−1.8	−0.9
$\Delta V^{s}$ (mL)	0.0	1.8	1.9
$C^{tot}$ (mL/mmHg)	1.1	1.5	1.1
$C^{s} /C^{tot}$ (%)	35	8	10
$1 - (C^{s} /C^{tot} )/ k_{V}$ (%)	0	77	71

### Compliance shift

As mentioned before, in upright posture the importance of cranial compliance increased for low CSF pressures. In upright equilibrium, only 10% of the total compliance were provided by the spinal compartment. This corresponded to a 71% reduction relative to the spinal compartment’s contribution in upright posture (Table 2). Under the condition of no change in total CSF volume after sitting up, the total compliance in upright posture strongly increased due to the steep increase in cranial compliance at low CSF pressure. Consequently, the contribution of the spinal compartment towards overall compliance became even lower.

Cerebral CSF pressure ($p_{CSF}^{c}$), total, cranial, and spinal change in CSF volume ($\Delta V^{tot}$, $\Delta V^{c}$, and $\Delta V^{s}$), total compliance ($C^{tot}$), and spinal compliance ($C^{s}$) in upright posture are shown in comparison to their reference values in horizontal position. The values were calculated under the two alternative assumed conditions of unchanged volume (Eq. 18) and unchanged total compliance (Eq. 19) relative to the horizontal position.

### Sensitivity

We analyzed the sensitivity of the reported results to changes in the nominal parameter values (Table 1). This nominal parameter set describes an NPH patient. The physiologic parameter set used to analyze the sensitivity of the model towards changes in the parameters $E$, $p_{1}$, $p_{0}$, and $R_{abs}^{tot}$ describes a subject with slightly lower CSF pressure in horizontal position. Also, the calculated cranial CSF pressure in upright posture was lower in the physiologic case (−6.6 mmHg after sitting up and −5.9 mmHg in upright equilibrium) compared to the NPH parameter set. The caudal shift of CSF volume caused by sitting up ($\Delta V^{s}$ in Table 2) was slightly higher (2.1 mL with the physiologic parameter set compared to 1.8 mL in the NPH case). The shift in compliance was not as pronounced as for the NPH parameter set, but the contribution of the spinal compartment to the total compliance still reduced to 18% in upright posture.

For a longer hydrostatic pressure column in the CSF system (*l*_*sc*_ = 36.3 cm), the effect of posture increased as the initial volume shift increased to 2.0 mL (not presented in Table 2), and the contribution of the spinal compliance in upright equilibrium decreased to 7%. Conversely, increased spinal venous pressure $p_{v}^{s}$ reduced the effect of the jugular vein collapse. Consequently, spinal compliance in upright equilibrium was still 12% and the initial CSF volume shift was reduced to 1.5 mL for 7.7 mmHg spinal venous pressure.

When using different values for the compliance contribution of the spinal compartment in horizontal position ($k_{V}$), the caudal shift in CSF volume changed almost proportionally. For example, $\Delta V^{s}$ reduced to 1.0 mL when $k_{V}$ was reduced by 50% ($k_{V} = 0.175$) and increased to 4.2 mL when $k_{V}$ was increased by 50% ($k_{V} = 0.525$). However, even for such large variations in the compliance distribution (±50%), the reduction of the relative spinal compliance remained between 70 and 82% of its value in horizontal position ($1 (C^{s} /C^{tot} )/ k_{V}$).

### Patent jugular veins

Without the collapse of the jugular veins (Eq. 7 instead of Eq. 8) only the difference in density can lead to shifts in CSF volume and compliance distribution when changing posture. In this modified model with patent jugular veins in upright posture, 0.4 mL of CSF flowed from the spinal into the cranial compartment when sitting up from horizontal. Cranial CSF pressure in upright posture decreased further (to −13.3 mmHg) with patent jugular veins compared to the case with collapsed jugular veins (−3.3 mmHg).

### Cardiac pulsations

**Fig. 3:**
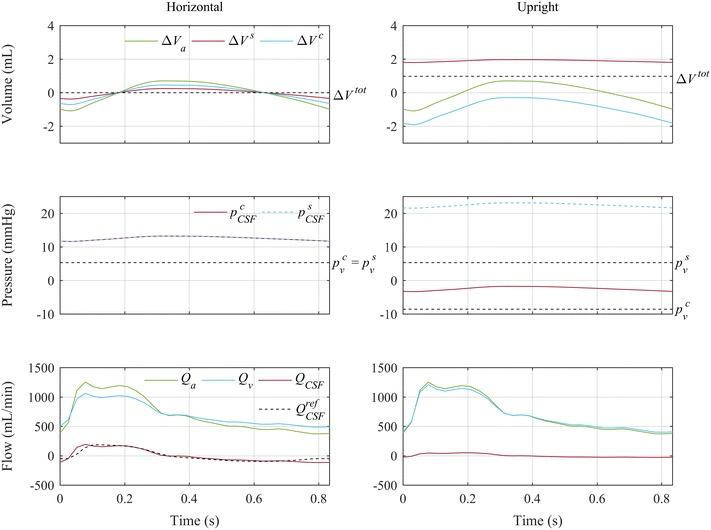
Effect of arterial pulsation in horizontal and upright posture. During the cardiac cycle, the cranial arterial inflow $Q_{a}$ (source [9]) leads to fluctuations in cranial arterial volume, $\Delta V_{a}$. Compensating craniospinal flows of CSF ($Q_{CSF}$) and venous blood ($Q_{v}$), craniospinal CSF distribution ($\Delta V^{c}$ versus $\Delta V^{s}$), and the corresponding cranial and spinal CSF pressures ($p_{CSF}^{c}$ and $p_{CSF}^{s}$, respectively) are shown for horizontal and upright posture. The corresponding measured CSF flow in supine position [9] is shown as *dashed line*. The flow rates are positive in the directions indicated in (Fig. 1)

The pulsatile arterial inflow $Q_{a}$ measured in NPH patients [9] caused a cranial arterial volume pulsation with 1.8 mL stroke volume (difference between maximum and minimum arterial volume, $\Delta V_{a}$). This change in cranial arterial volume was compensated by craniospinal flows of CSF and venous blood with 0.6 mL and 1.2 mL stroke volume, respectively (Fig. 3, left column). This stroke volume of the craniospinal CSF flow was 35% of the arterial stroke volume, which corresponds to the analytical value of $k_{V}$. The total CSF volume hardly changed during one cardiac cycle (less than 1 µL) due to negligible variations in CSF absorption during that short time frame. Nevertheless, the cardiac pulsations caused substantial CSF pressure amplitudes (1.6 mmHg). The pulsation of the spinal CSF volume was directly proportional to the arterial waveform. By definition (Eq. 21), the remaining portion of the pulsatile arterial blood flow was compensated by fluctuations of craniospinal venous blood flow rate $Q_{v}$.

In upright posture, this picture changed (Fig. 3, right column). While no change in arterial blood flow was prescribed, craniospinal CSF stroke volume was nevertheless reduced to 10% of the arterial stroke volume (0.2 mL). However, despite these changes in fluid dynamics and changes in absolute pressures, CSF pulse pressure amplitudes remained constant at 1.6 mmHg.

**Fig. 4:**
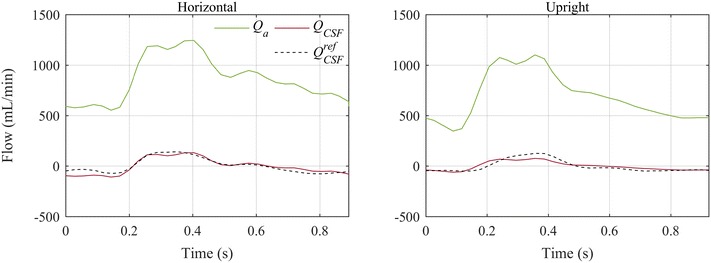
Comparison of simulated to measured CSF flow pulsation in horizontal and upright posture. Craniospinal CSF flow rates ($Q_{CSF}$) were simulated in supine and upright posture, based on arterial inflow measured by Alperin et al. [2] in supine and sitting posture. The corresponding measured CSF pulsations are plotted as *dashed lines*

The simulated craniospinal CSF flow pulsations of a healthy subject in horizontal and upright position (Fig. 4) differed from the corresponding measurements in supine and sitting posture [2] by a mean absolute error of 22 and 21 mL/min, respectively. The physiological parameter set (*E* = 0.1/mL, *p*_1_ = 10 mmHg, *p*_0_ = −5.3 mmHg, $R_{abs}^{tot} = 13.4\, {\text{mmHg}}/({\text{mL}}/\hbox{min} )$) was used for these simulations.

## Discussion

### Volume and compliance shifts

Our model predicts a posture-dependent shift of the craniospinal compliance distribution caused by a caudal displacement of CSF volume. As previously observed by Magnaes [1], this CSF volume displacement in upright posture reduces the compliance provided by the spinal compartment including the spinal thecal sac. It is induced by the hydrostatic pressure column, which is greater in the CSF system compared to the veins, where it is interrupted by the collapsing jugular veins. The estimated shifts of CSF volume and compliance are in range of the observations of Magnaes [1], although he assumed a much higher contribution of the spinal compartment to compliance than in this study [17]. Furthermore, the posture-dependent shift of the craniospinal compliance distribution was also observed for large variations of the employed parameter values, indicating that our analysis is robust.

### Jugular collapse

Without collapsing jugular veins, the model showed neither a caudal shift of CSF volume nor a cranial shift of the compliance distribution. Furthermore, the fall in cranial CSF pressure was greater than that observed clinically [4, 5]. As jugular collapse reduces this fall in pressure in upright posture, the jugular veins may be seen as serving a protective function for the brain. In hydrocephalus patients with ventriculoperitoneal or ventriculoatrial shunts, this protective mechanism is partially bypassed so that, without appropriate siphon prevention, ICP can decrease to levels as low as those predicted by our model without jugular collapse.

### Pressure–volume relationship

The exponential pressure–volume relationship of the CSF system is well proven, at least for normal and reasonably increased CSF pressures (relative to the sagittal sinus pressure). However, for sufficiently decreased CSF volume, it implies infinite compliance. This attribute of the exponential pressure–volume relationship becomes especially problematic when applied to the cranial compliance in upright posture, because negative CSF to venous pressure gradients could easily be reached here. However, as such gradients were not reached in this study this limitation does not affect the results or conclusions reported herein. Nonetheless, extrapolation to low CSF pressures would be invalid (Fig. 2). Therefore, a more accurate description of the pressure–volume relationships would need to be used to study the effect of shunting on CSF dynamics [16, 21].

Only considering the mean venous pressure as counter-pressure for the pressure–volume relationship might seem simplistic, as venous pressure varies over the different generations of venous vessels. However, the implemented pressure–volume relationship captures this venous pressure variation and distribution with its exponential shape [22].

The only mechanism of compliance included in the model is the displacement of venous blood. While this mechanism is accepted as the main contributor to compliance in the cranium [10], this is less clear for the rest of the craniospinal space, especially for the spinal thecal sac. However, due to the high distensibility of venous vessels [23], tissue pressure strongly correlates with venous pressure throughout the body. Therefore, it is reasonable to assume that venous pressure is the relevant counter-pressure to compliance in the entire CSF system. If the surrounding tissue itself could provide elastic recoil, part of the pressure–volume relation would have to be modelled independent of venous pressure. This would only then decrease the modelled compliance shift, if the elastic tissue were located intracranially, since the counter-pressure of the spinal compartment is already assumed to be independent of posture due to its proximity to the venous hydrostatic indifference point [4].

### CSF absorption

Before Magnaes [1] determined the craniospinal compliance distribution in some of his patients, similar experiments had been done in adult cats [7]. While in cats the spinal compartment appeared to be less important for compliance, it was still responsible for a significant portion of CSF absorption (16%). Similar proportions of the craniospinal CSF absorption distribution were predicted by our model under the condition of posture-independent total compliance. While this result supports the hypothesis that there is spinal CSF absorption, the exact proportion predicted by the model is sensitive to the employed equilibrium pressure in upright posture. Furthermore, the lengths of hydrostatic pressure columns were calculated based on the assumption of 100% cranial absorption [4]. Taking spinal absorption into account, the estimated value of $l_{jug}$ would slightly increase, which would decrease the compliance shift predicted by our model.

### Craniospinal flows

Pulsatile arterial inflow into the cranium were compensated by simultaneous craniospinal outflow of CSF and venous blood. Stroke volume and amplitude of the calculated CSF pulsations (Figs. 3, 4) were very close to the respective values measured in vivo [2, 8, 9]. Even the reduction in CSF stroke volume was predicted well (Fig. 4). These are strong indications that the increased resistance of the jugular veins in upright posture is responsible for the shift in compliance observed in vivo. Jugular vein collapse can thus be considered a major contributor to CSF dynamics in upright posture. The calculated overall CSF volume hardly changed within a cardiac cycle. Therefore, the ratio of the estimated CSF and the applied arterial stroke volumes was equal to the contribution of the spinal compartment to the overall compliance. In MRI measurements, CSF and even more so the venous pulses are delayed compared to the arterial input. At least some of this delay can be attributed to wave propagation due to vascular distensibility [23]. In the model, these phase shifts between the calculated craniospinal waves were ignored with the implicit assumption of instantaneous transmission of pressures throughout the craniospinal space. However, when assuming that most of the phase shift originates from a wave propagation delay, it does not influence the compliance distribution estimated from the ratio of CSF and arterial stroke volume. In addition to being delayed, recorded venous pulsations appear damped compared to the modelled pulsations. This damping is probably caused by the Windkessel effect in the larger veins, which is not included in our model. However, the craniospinal venous flow rate is not only difficult to model, it is also difficult to measure with MRI as, especially in upright posture it is distributed over numerous small vessels.

## Conclusion

Our results support the hypothesis that the jugular veins play an important role in posture-related changes of CSF dynamics, as their collapse in upright posture induces substantial changes in CSF pressure and compliance.

## Abbreviations

CSF: cerebrospinal fluid
ICP: intracranial pressure
MRI: magnet-resonance imaging
NPH: normal pressure hydrocephalus
